# Associations and pathways between residential greenness and metabolic syndromes in Fujian Province

**DOI:** 10.3389/fpubh.2022.1014380

**Published:** 2022-12-22

**Authors:** Xiaoqing Li, Qinjian Wang, Chuanteng Feng, Bin Yu, Xi Lin, Yao Fu, Shu Dong, Ge Qiu, Darren How Jin Aik, Yanrong Yin, Pincang Xia, Shaofen Huang, Nian Liu, Xiuquan Lin, Yefa Zhang, Xin Fang, Wenling Zhong, Peng Jia, Shujuan Yang

**Affiliations:** ^1^Department for Chronic and Noncommunicable Disease Control and Prevention, Fujian Provincial Center for Disease Control and Prevention, Fuzhou, China; ^2^West China School of Public Health and West China Fourth Hospital, Sichuan University, Chengdu, China; ^3^Institute for Disaster Management and Reconstruction, Sichuan University, Chengdu, China; ^4^School of Resource and Environmental Sciences, Wuhan University, Wuhan, China; ^5^International Institute of Spatial Lifecourse Health (ISLE), Wuhan University, Wuhan, China; ^6^Department for HIV/AIDS and STDs Control and Prevention, Fujian Provincial Center for Disease Control and Prevention, Fuzhou, China

**Keywords:** greenness, normalized difference vegetation index (NDVI), enhanced vegetation index (EVI), metabolic syndrome (MetS), independent mediation effect, joint mediation effect

## Abstract

**Background:**

Greenness exposure is beneficial to human health, but its potential mechanisms through which the risk for metabolic syndrome (MetS) could be reduced have been poorly studied. We aimed to estimate the greenness-MetS association in southeast China and investigate the independent and joint mediation effects of physical activity (PA), body mass index (BMI), and air pollutants on the association.

**Methods:**

A cross-sectional study was conducted among the 38,288 adults based on the Fujian Behavior and Disease Surveillance (FBDS), established in 2018. MetS was defined as the presence of three or more of the five components: abdominal obesity, elevated triglyceride, reduced high-density lipoprotein cholesterol (HDL-C), high blood pressure, and elevated fasting glucose. The residential greenness exposure was measured as the 3-year mean values of the normalized difference vegetation index (NDVI) and enhanced vegetation index (EVI) within the 250, 500, and 1,000 meters (m) buffer zones around the residential address of each participant. Logistic regression models were used to estimate the greenness-MetS association. The causal mediation analysis was used to estimate the independent and joint mediation effects of PA, BMI, particulate matter with an aerodynamic diameter of 2.5 μm (PM_2.5_), particulate matter with an aerodynamic diameter ≤ 10 μm (PM_10_), nitrogen dioxide (NO_2_), and sulfur dioxide (SO_2_).

**Results:**

Each interquartile range (*IQR*) increase in greenness was associated with a decrease of 13% (*OR* = 0.87 [95%*CI*: 0.83, 0.92] for NDVI_500m_ and *OR* = 0.87 [95%*CI*: 0.82, 0.91] for EVI_500m_) in MetS risk after adjusting for covariates. This association was stronger in those aged < 60 years (e.g., *OR* = 0.86 [95%*CI*: 0.81, 0.92] for NDVI_500m_), males (e.g., *OR* = 0.73 [95%*CI*: 0.67, 0.80] for NDVI_500m_), having an educational level of primary school or above (*OR* = 0.81 [95%*CI*: 0.74, 0.89] for NDVI_500m_), married/cohabitation (*OR* = 0.86 [95%*CI*: 0.81, 0.91] for NDVI_500m_), businessman (*OR* = 0.82 [95%*CI*: 0.68, 0.99] for NDVI_500m_), other laborers (*OR* = 0.77 [95%*CI*: 0.68, 0.88] for NDVI_500m_), and non-smokers (*OR* = 0.77 [95%*CI*: 0.70, 0.85] for NDVI_500m_). The joint effect of all six mediators mediated about 48.1% and 44.6% of the total effect of NDVI_500m_ and EVI_500m_ on the MetS risk, respectively. Among them, BMI showed the strongest independent mediation effect (25.0% for NDVI_500m_), followed by NO_2_ and PM_10_.

**Conclusion:**

Exposure to residential greenness was associated with a decreased risk for MetS. PA, BMI, and the four air pollutants jointly interpreted nearly half of the mediation effects on the greenness-MetS association.

## Introduction

Metabolic syndrome (MetS) is considered a primary cause of death and disability globally, especially in low- and middle-income countries (LMICs) (1). MetS consists of interrelated risk factors of cardiovascular diseases (CVDs), including dysglycemia, raised blood pressure, elevated triglyceride levels, low high-density lipoprotein cholesterol levels, and obesity (particularly central adiposity) (2). People with MetS are at twice the risk of developing CVD over the next 5 to 10 years than those without MetS. The increasing evidence has suggested an array of risk factors of MetS, such as a low level of physical activity (PA), weight gain, and an unhealthy diet (3).

Emerging evidence is beginning to link the upstream determinants, such as environmental factors (e.g., air pollution and residential greenness), to interrelated risk factors of MetS. For example, air pollution has been associated with obesity (4), as a high concentration of air pollutants may lead to metabolic dysfunctions through increased oxidative stress and inflammations in adipose tissue (5), accumulations of hepatic lipid (6), and the reduction of glucose utilization in skeletal muscle (7). Air pollution may also increase the risk for other chronic diseases, such as cardiovascular disease, respiratory disease, and cancer, thus further indirectly affect body weight (8). Also, environment can affect the risk for MetS through influencing their behaviors. For example, poor air quality may prevent people from engaging in regular PA and encourage sedentary behavior (9), and a green and human attractive residential environment may promote walking and cycling (10–12), which can all change the level of risk factors of MetS. Therefore, environmental factors may also be crucial for MetS.

Accumulating evidence has suggested that greenness exposure could benefit human health, including providing more opportunities for PA and social activities, reducing feelings of stress, and mitigating exposure to air pollution (13, 14). These factors may have an independent or a combined effect on the pathogenesis of MetS. For example, a study in Liaoning Province of China has shown that only particulate matter with an aerodynamic diameter of ≤ 2.5 μm (PM_2.5_), nitrogen dioxide (NO_2_), and ozone (O_3_) mediated the association between greenness and MetS risk (15). At the same time, another study in the UK has shown that the greenness-MetS association may be mediated by PA and air pollution exposure (16). These existing studies have only examined the independent mediation effects of those potential mediators, which usually co-exist and may interact with one another to complicate their health effects. The multiple mediation analysis that investigates the joint effect may be of great significance to yield an accurate estimate (17). Hence, it is necessary to investigate the joint effect of those mediators between greenness exposure and MetS risk, which can better understand potential mechanisms underlying residential greenness and MetS risk.

Globally, China has the highest burden of CVDs and is facing a CVD crisis. Deaths from CVDs in China have nearly doubled in the past few decades, while rates are falling in other high-income countries (18). Hence, China has the most urgent need to study MetS for early CVD prevention (19). This study aimed to estimate the associations between long-term exposure to greenness and MetS risk in China and investigate the independent and joint mediation effects of air pollutants, PA, and body mass index (BMI) on the greenness-MetS association. The findings would serve as solid evidences for intervention design and policy-making to further reduce MetS risk.

## Materials and methods

### Study design and dataset

This study was based on the Fujian Behavior and Disease Surveillance (FBDS), a cross-sectional study investigating health-related behaviors and chronic diseases among adults in Fujian Province, China. The baseline dataset of the FBDS, investigated from June 2018 to December 2020, was used in this study.

A multi-stage, stratified cluster random sampling method was adopted to recruit FBDS participants from communities. The eight prefecture-level cities in Fujian Province were considered as the first sampling level. About 3–6 districts (urban) or counties (rural) were selected from each prefecture-level city. 4–6 urban subdistricts or rural townships were selected with probability proportional to size from each district/county. Then, 3–5 neighborhood communities or administrative villages were selected with probability proportional to size from each subdistrict/township. Subsequently, Each community or village was devided into several groups with the size of 50 households and 2 groups were randomly selected from each community or village. All 50 households in each group were clusterly sampled. Finally, one adult aged ≥ 18 years was selected from every household by a Kish selection table. The selected household that can not respond to all three attempts made on 3 days was replaced by another household with a similar family structure (e.g., nuclear family) in the same village or residential area. Medical staff in township health centers phoned and briefed prospective participants about the study and confirmed their eligibility to participate in the study.

The inclusion criteria for FBDS participants were (1) age ≥ 18 years on the day of the investigation; (2) permanent residents living in their current residence for at least 6 months, the capability of completing baseline surveys; and (3) complete questionnaire interviews, physical examinations, and blood tests. As for the exclusion criteria, they were (1) severe mental diseases (e.g., schizophrenia and bipolar disorder), and (2) unable to communicate with the interviewers. A total of 54,961 adults aged 18 years or older from 212 rural townships or urban subdistricts were invited to our survey. Upon arrival at the survey sites, participants who voluntarily decided to take part in the study were again informed about the study by the trained interviewers, and completed a written consent form. All participants received medical examinations and clinical laboratory tests (Supplementary Figure S1). In the current analysis, we excluded 16,673 individuals who had incomplete information on MetS and its components. Finally, a total of 38,288 adults were included in this study (Supplementary Figure S2).

The Ethical Review Board of the Fujian Provincial Center for Disease Control and Prevention reviewed and approved the study protocol (2018001).

### Outcome variables

The MetS was assessed according to the criteria for diagnosis by the National Heart, Lung, and Blood Institute, American Heart Association, World Heart Federation, International Atherosclerosis Society, and International Association for the Study of Obesity in 2009 (2). It was defined as the presence of three or more of the following five components: (1) abdominal obesity, described as waist circumference ≥ 90 cm for men and ≥ 85 cm for women (20); (2) elevated triglyceride, defined as a fasting triglyceride level ≥ 1.7 mmol/L (150 mg/dL) or the reported receipt of elevated triglyceride medication (21); (3) reduced high-density lipoprotein cholesterol (HDL-C), referring to a level < 1.03 mmol/L (<40 mg/dL) in men and 1.29 mmol/L (<50 mg/dL) in women, or the reported receipt of reduced HDL-C medication (21); (4) high blood pressure, referring to systolic 130 or diastolic 85 mmHg or any use of antihypertensive medication; and (5) elevated fasting glucose, defined as a fasting plasma glucose ≥ 5.6 mmol/L or the reported receipt of glucose-lowering medication (22).

### Exposure variables

Residential greenness was measured by the satellite-based normalized difference vegetation index (NDVI) and enhanced vegetation index (EVI), derived from satellite imagery. Both vegetation indices were derived from the MOD13Q1.061 Terra Vegetation Indices 16-Day Global 250 m SIN Grid, computed at a spatial resolution of 250 m by surface reflectance from the Moderate-resolution Imaging Spectroradiometer (MODIS) satellite images of the National Aeronautics and Space Administration's (NASA) (23). NDVI is calculated based on satellite sensors' red and near-infrared bands (24–26). EVI is calculated similarly to NDVI but takes an additional blue light band to correct the atmospheric and soil background for enhanced accuracy. Hence, EVI has been considered more sensitive than NDVI in high-biomass areas (24–26). Both NDVI and EVI values range from −1 to 1, where higher positive value indicates a higher coverage of greenness; in contrast, negative values represent other features with high surface reflectance, such as clouds, water, and snow. We processed and analyzed Landsat 8 Thematic Mapper satellite images with a minimum of cloud cover for generating NDVI/EVI. We defined residential greenness as the annual mean NDVI/EVI during 2016–2018 (the 3 years before the survey) within the 250, 500, and 1,000 m buffer zones around the residential address of each participant, for easier comparison with similar studies (27, 28). The 500 m radius of the buffer zone has been widely used in many similar studies to measure residential greenness (29), so the results from the 500 m buffer zone were presented as the main results.

### Mediators

We identified common potential confounding variables between greenness exposure and MetS risk from the available literature, and incorporated them in a directed acyclic graph (DAG) to select a minimal set of covariates (30). PA, BMI, and air pollution exposure appeared in the DAG as mediators between greenness and MetS (Supplementary Figure S3), as shown in the previous literature (15, 16, 31, 32). PA was measured by the days of conducting physical activity per week. BMI was calculated as one's body weight (kg) divided by the squared height (m^2^). The exposures to PM_2.5_, PM_10_, NO_2_, and sulfur dioxide (SO_2_) were calculated as the mean concentrations over the residential address of each participant during the 3 years before the survey. The concentration of the air pollutants was estimated at a 1-km spatial resolution by random forest models, on the basis of the observation data from 1,480 on-ground monitoring stations, satellite data, meteorological factors (temperature and humidity), land use information, and other spatial and temporal predictors (33). A 10-fold cross-validation process was performed to assess the validity of the predictions for PM_2.5_ (the overall adjusted R^2^ = 0.84, the Root Mean Square Error = 9.24), PM_10_ (0.77, 18.91), NO_2_ (0.79, 4.94), and SO_2_ (0.70, 3.67).

### Covariates

According to the DAG (Supplementary Figure S3), three major groups of covariates were controlled for in this study, including sociodemographic characteristics, lifestyle habits, and environmental factors. The sociodemographic characteristics included age (year), sex (male or female), ethnicity (Han or minority), and educational level (little or no formal education, primary school, junior or high school, or bachelor's degree or above). Additionally, marriage status (married/cohabitation, unmarried, or widowed/divorced/separated), occupation (unemployed, farmer, businessman, administrative staff, student, or other laborers), insurance type (commercial insurance, non-commercial insurance, or no insurance), and residential location (urban or rural) were chosen. Lifestyle habits included smoking status (non-smokers, or current or former smokers), second-hand smoking (no or yes), alcohol drinking status (non-drinkers, or current or former drinkers), and dietary habits (days per month for grains, vegetables, meat, fruits, aquatic products, and milk). As for the environmental factors, temperature (°C) and humidity (%) were selected. Subsequently, the annual temperature and humidity over the participants' residential addresses for the three years before the survey were averaged; this data was obtained from the China Meteorological Administration (34).

### Statistical analyses

The primary characteristics of the participants were summarized by proportions or means with standard deviations (*SD*). We used logistic regression models to assess the associations between long-term exposure to greenness and MetS and its related indexes. The results are presented as odds ratios (*OR*s) with corresponding 95% confidence intervals (*CI*s) for each interquartile range (*IQR*) increment in NDVI and EVI after adjusting for covariates. To assess the robustness of our results, we conducted the following sensitivity analyses: (1) estimating the association between greenness exposure and MetS risk at the buffer areas of 250 and 1,000 m; (2) fitting the adjusted model using the one to three years average greenness value to test the robustness of the effect estimations.

We also performed a stratified analysis. It was categorized by age, sex, ethnicity, educational level, marriage status, occupation, insurance type, drinking status, smoking status, and residence to examine the associations across different sub-populations. Subsequently, the interaction between stratified factors and greeness exposure were performed and presented as a cross-product term in the regression model (i.e., NDVI ^*^ age or NDVI ^*^ sex or NDVI ^*^ ethnicity).

Based on the DAG, PA, BMI, PM_2.5_, PM_10_, NO_2_, and SO_2_ were considered the mediators in the association between greenness exposure and MetS, and their independent and combined mediation effects were examined by causal mediation analysis (17, 35). Individual effects were tested by including each mediator separately in the full model, while for the joint effect of multiple mediators, a set of mediators (PA, BMI, PM_2.5_, PM_10_, NO_2_, and SO_2_) was included jointly in the full model, regardless of order. Mediation analysis, based on the counterfactual view, was a regression-based multiple mediation approach to considering multiple mediators simultaneously, which can also be adapted to situations within which mediators interact with one another (17, 35, 36). In this approach, we used regression models including residential greenness, PA, BMI, PM_2.5_, PM_10_, NO_2_ and SO_2_, and covariates. Next, we used six ordinal logistic regressions to regress PA, BMI, PM_2.5_, PM_10_, NO_2_, and SO_2_ on residential greenness and covariates (37, 38). These analyses were performed using the function “CMA verse”, implemented in the R package (35). The total effect can be decomposed into direct (not through mediators) and indirect/mediation effects (through mediators). Finally, for each mediator and the joint six mediators, we calculated the indirect/mediation effect percentage over the total impact. The standard errors of the mediation effect was estimated by generating 5000 bootstrap iterations.

The DAG was constructed in DAGitty (v3.0). All statistical analyses were analyzed in R Foundation for Statistical Computing (version 4.0.3). We had set the significance threshold to 0.05, and all analysis tests were 2-sided.

## Results

### Baseline characteristics

The study participants' average (*SD*) age was 53.63 (13.65) years, and 52.77% were women. The majority of participants were of Han ethnicity (98.51%), unemployed and farmer (62.07%), and had an educational level below primary school (80.03%) (Table 1). Those participants who were females, elderly, widowed/divorced/separated, and rural residents, had lower educational level, a habit of never smoking and alcohol drinking, higher BMI, and a lower intake of cereal, vegetables, meat, fruits, aquatic products, and milk, and exercise less regularly were more likely to have MetS (all *P*-value<0.05). The mean (*SD*) of the 3-year average air pollution concentrations were 29.11 μg/m^3^ (2.66 μg/m^3^), 51.86 μg/m^3^ (5.82 μg/m^3^), 21.35 μg/m^3^ (2.89 μg/m^3^), and 8.96 μg/m^3^ (1.76 μg/m^3^) for PM_2.5_, PM_10_, NO_2_, and SO_2_, respectively.

**Table 1:** Characteristics of the study participants.

**Variables**	**Percentage (%) or mean** ±**SD**			***P*-value^a^**
	**Total**	**With MetS**	**Without MetS**	
	***N* = 38,288 (%)**	***N* = 9,021 (%)**	***N* = 29,267 (%)**	
**Sociodemographics**				
Age (year, IQR)				
[18,30)	1,754 (4.59)	135 (1.50)	1,619 (5.55)	< 0.001
[30,40)	4,410 (11.55)	513 (5.70)	3,897 (13.35)	
[40,50)	8,050 (21.07)	1,352 (15.02)	6,698 (22.94)	
[50,60)	23,984 (62.79)	7,001 (77.78)	16,983 (58.16)	
Sex				
Male	18,082 (47.23)	3,750 (41.57)	14,332 (48.97)	< 0.001
Female	20,206 (52.77)	5,271 (58.43)	14,935 (51.03)	
Ethnicity				
Han	37,719 (98.51)	8,879 (98.43)	28,840 (98.54)	0.430
Minority	569 (1.49)	142 (1.57)	427 (1.46)	
Educational level				
little or no formal education	13,036 (34.05)	3,902 (43.25)	9,134 (31.21)	< 0.001
Primary school	17,605 (45.98)	3,811 (42.25)	13,794 (47.13)	
Junior or high school	4,735 (12.36)	958 (10.62)	3,777 (12.91)	
Bachelor degree or above	2,912 (7.61)	350 (3.88)	2,562 (8.75)	
Marriage status				
Married/Cohabitation	33,866 (88.45)	7,644 (84.74)	26,222 (89.59)	< 0.001
Unmarried	1,523 (3.98)	236 (2.62)	1,287 (4.40)	
Widowed/Divorced/Separated	2,899 (7.57)	1,141 (12.64)	1,758 (6.01)	
Occupation				
Unemployed	11,959 (31.23)	3,446 (38.20)	8,513 (29.09)	< 0.001
Famer	11,807 (30.84)	2,689 (29.81)	9,118 (31.15)	
Businessman	4,332 (11.31)	711 (7.88)	3,621 (12.37)	
Administrative staff	1,977 (5.16)	393 (4.36)	1,584 (5.41)	
Student	212 (0.55)	19 (0.21)	193 (0.66)	
Other laborers	8,001 (20.91)	1,763 (19.54)	6,238 (21.32)	
Insurance type				
Commercial insurance	1,574 (4.11)	263 (2.92)	1,311 (4.48)	< 0.001
Non-commercial insurance	36,322 (94.87)	8,663 (96.03)	27,659 (94.51)	
No insurance	392 (1.02)	95 (1.05)	297 (1.01)	
Residential location				
Urban	7,459 (19.48)	1,472 (16.32)	5,987 (20.46)	< 0.001
Rural	30,829 (80.52)	7,549 (83.68)	23,280 (79.54)	
**Lifestyle behaviors**				
Smoking status				
Non-smokers	26,649 (69.60)	6,518 (72.25)	20,131 (68.78)	< 0.001
Current or former smokers	11639 (30.40)	2503 (27.75)	9136 (31.22)	
Second-hand smoking				
No	20,492 (53.52)	5,131 (56.88)	15,361 (52.49)	< 0.001
Yes	17,796 (46.48)	3,890 (43.12)	13,906 (47.51)	
Alcohol drinking status				
Non-drinkers	24,849 (64.90)	6,148 (68.15)	18,701 (63.90)	< 0.001
Current or former drinkers	13,439 (35.10)	2,873 (31.85)	10,566 (36.10)	
Dietary habits (days/month)				
Grains	25.18 (±3.96)	25.13 (±4.14)	25.20 (±3.90)	0.420
Vegetables	25.11 (±3.76)	24.87 (±4.30)	25.19 (±3.57)	< 0.001
Meat	18.67 (±9.02)	17.69 (±9.44)	18.98 (±8.87)	< 0.001
Fruits	13.79 (±9.93)	12.30 (±9.86)	14.25 (±9.90)	< 0.001
Aquatic products	11.87 (±9.87)	10.46 (±9.81)	12.30 (±9.85)	< 0.001
Milk	5.61 (±9.00)	4.63 (±8.41)	5.92 (±9.15)	< 0.001
Physical activity				
≤ 3 days/week	23,457 (61.26)	5,656 (62.70)	17,801 (60.82)	0.001
>3 days/week	14,831 (38.74)	3,365 (37.30)	11,466 (39.18)	
BMI, kg/m^2^	24.39 (±3.33)	25.56 (±3.53)	24.03 (±3.18)	< 0.001
**Environmental factors**				
Air pollution				
PM_2.5_ (μg/m^3^)	29.11 (±2.66)	28.99 (±2.46)	29.15 (±2.71)	< 0.001
PM_10_ (μg/m^3^)	51.86 (±5.82)	51.65 (±5.56)	51.93 (±5.89)	0.006
NO_2_ (μg/m^3^)	21.35 (±2.89)	21.42 (±2.84)	21.33 (±2.91)	< 0.001
SO_2_ (μg/m^3^)	8.96 (±1.76)	8.94 (±1.69)	8.97 (±1.78)	0.610
Temperature (°C)	20.18 (±1.24)	20.04 (±1.27)	20.22 (±1.22)	< 0.001
Humidity (%)	79.18 (±2.03)	79.21 (±2.12)	79.17 (±2.00)	0.290
**Mets related components**				
Central obesity				
Yes	195 (0.51)	118 (1.83)	65 (0.22)	< 0.001
No	38,093 (99.49)	6,324 (98.17)	29,202 (99.78)	
Elevated triacylglyceride				
Yes	14,837 (38.75)	7,875 (87.30)	6,962 (23.78)	< 0.001
No	23,451 (61.25)	1,145 (12.70)	22,306 (76.22)	
Reduced HDL-cholesterol				
Yes	10,579 (27.63)	6,084 (67.48)	4,495 (15.36)	< 0.001
No	27,709 (72.37)	2,933 (32.52)	24,776 (84.64)	
High blood pressure				
Yes	20,174 (52.69)	8,157 (90.53)	12,017 (41.05)	< 0.001
No	18,114 (47.31)	855 (9.47)	17,259 (58.95)	
Raised fasting glucose				
Yes	13,382 (34.95)	6,899 (76.90)	6,482 (22.11)	< 0.001
No	24,906 (65.05)	2,072 (23.10)	22,835 (77.89)	
Greenness^b^ **(%)**				
NDVI_250m_	0.51 (±0.17)	0.51 (±0.17)	0.50 (±0.17)	0.063
NDVI_500m_	0.54 (±0.17)	0.55 (±0.18)	0.54 (±0.17)	0.005
NDVI_1000m_	0.57 (±0.17)	0.58 (±0.17)	0.57 (±0.17)	< 0.001
EVI_250m_	0.37 (±0.13)	0.37 (±0.13)	0.37 (±0.13)	0.830
EVI_500m_	0.40 (±0.13)	0.40 (±0.13)	0.40 (±0.13)	0.280
EVI_1000m_	0.42 (±0.15)	0.43 (±0.14)	0.42 (±0.16)	0.005

BMI, body mass index; EVI, enhanced vegetation index; IQR, interquartile range; NDVI, normalized difference vegetation index; NO_2_, nitrogen dioxide; PM_2.5_, particulate matter with an aerodynamic diameter of 2.5 μm; PM_10_, particulate matter with an aerodynamic diameter ≤ 10 μm; SO_2_,sulfur dioxide.

a *P*-values tested the significance of differences in each variable between with and without MetS participants and were based on χ^2^-test for categorical variables or *t*-test for continuous variables.

b The means of annual NDVI and EVI within the 250, 500, and 1,000 m buffer zones around the participants' residential addresses during the 3 years before the survey.

**Figure 1:**
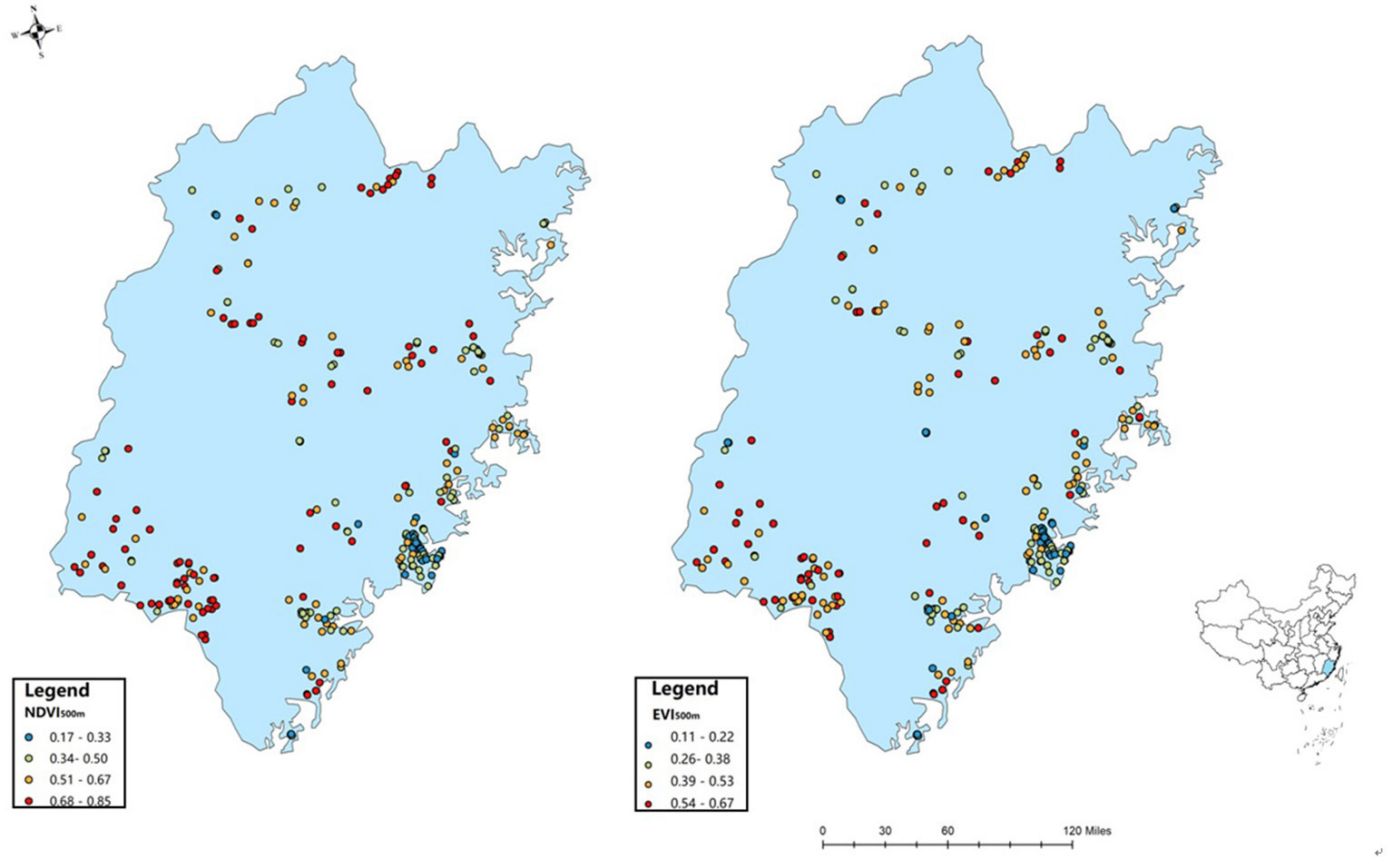
Residential greenness exposure and location of the study area on the map of China. NDI, normalized difference vegetation index; EVI, enhanced vegetation index.

Moreover, there was spatial heterogeneity in levels of greenness exposure among the study communities (Figure 1). For example, the range of the NDVI_500m_ and EVI_500m_ were 0.16–0.90 and 0.11–0.73, respectively. Hence, the NDVI and EVI values were highly correlated, with *r*^2^ ranging from 0.86 to 0.97 (Supplementary Figure S4).

### Greenness exposure and MetS risk

In the crude model, higher greenness exposure at NDVI_500m_ and EVI_500m_ was significantly associated with a higher risk of MetS (Table 2). However, when adjusted to our covariates model, each *IQR* increase in NDVI_500m_ and EVI_500m_ was associated with a 13% reduced risk of MetS after adjusting for covariates (NDVI_500m_: *OR* = 0.87 [95% *CI*: 0.83, 0.92]; EVI_500m_: *OR* = 0.87 [0.82, 0.91]), respectively (Table 2).

**Table 2:** Associations of each *IQR* increase residential greenness exposure in NDVI_500m_ and EVI_500m_ with the MetS risk.

**MetS related indexes**	***OR*** **(95%** ***CI*****) for NDVI**_**500m**_		***OR*** **(95%** ***CI*****) for EVI**_**500m**_	
	**Crude model**	**Adjusted model^a^**	**Crude model**	**Adjusted model^a^**
MetS	1.08 (1.03, 1.12)^**^	0.87 (0.83, 0.92)^**^	1.03 (0.99, 1.08)	0.87 (0.82, 0.91)^**^
Central obesity	0.55 (0.42, 0.73)^**^	0.66 (0.48, 0.91)^*^	0.64 (0.48, 0.84)^**^	0.76 (0.56, 1.04)
Elevated triacylglyceride	1.07 (1.03, 1.11)^**^	0.93 (0.89, 0.98)^*^	1.01 (0.98, 1.05)	0.91 (0.87, 0.95)^**^
Reduced HDL-cholesterol	1.07 (1.03, 1.12)^**^	1.04 (0.99, 1.09)	1.08 (1.03, 1.12)^**^	1.06 (1.01, 1.11)^*^
High blood pressure	1.24 (1.20, 1.29)^**^	0.97 (0.93, 1.02)	1.20 (1.16, 1.25)^**^	0.96 (0.92, 1.00)
Raised fasting glucose	0.85 (0.82, 0.88)^**^	0.68 (0.65, 0.71)^**^	0.83 (0.79, 0.86)^**^	0.69 (0.66, 0.72)^**^

EVI, enhanced vegetation index; *IQR*, interquartile range; MetS, metabolic syndrome; NDVI, normalized difference vegetation index.

a Adjusted for age, sex, ethnicity, educational level, occupation, marriage status, residential location, diet, smoking status, alcohol drinking status, temperature, and humidity.

* *P*-value < 0.05;

** *P*-value < 0.01.

We observed that each *IQR* increase in NDVI_500m_ was associated with a decreased risk for central obesity (*OR* = 0.66 [0.48, 0.91]), elevated triacylglyceride (*OR* = 0.93 [0.89, 0.98]) and raised fasting glucose (*OR* = 0.68 [0.65, 0.71]). While, an reduced risk of elevated triacylglyceride (*OR* = 0.91 [0.87, 0.95]) and raised fasting glucose (*OR* = 0.69 [0.66, 0.72]) was observed for EVI_500m_. However, we found an increased risk of reduced HDL-cholesterol (*OR* = 1.06 [1.01, 1.11]) for EVI_500m_ (Table 2).

Each *IQR* increase in EVI and NDVI at the buffer areas of 250m and 1,000m significantly reduced the MetS risk (NDVI_250m_: *OR* = 0.89 [0.84, 0.93]; EVI_250m_: *OR* = 0.88 [0.84, 0.92]; NDVI_1000m_: *OR* = 0.89 [0.84, 0.94]; EVI_1000m_: *OR* = 0.89 [0.85, 0.94]), and the results were similar with EVI at the buffer of 500 m (Supplementary Tables S1, S2). Moreover, the annual mean of greenness exposure over the past 1-, 2- and 3-years showed the robust effect estimates for MetS risk (Supplementary Table S3).

In stratified analysis, stronger associations between greenness exposure in NDVI_500m_ and EVI_500m_ and MetS risk were observed in young, male, having an educational level of primary school or above, married/cohabitation, businessman and non-smokers subgroups. For example, those aged <60 years (NDVI_500m_: *OR* = 0.86 [0.81, 0.92]; EVI_500m_: *OR* = 0.85 [0.79, 0.90]), males (NDVI_500m_: *OR* = 0.73 [0.67, 0.80]; EVI_500m_: *OR* = 0.74 [0.68, 0.81]), those with an educational level of primary school or above (NDVI_500m_: *OR* = 0.81 [0.74, 0.89]; EVI_500m_: *OR* = 0.84 [0.76, 0.92]), married/cohabitation participants (NDVI_500m_: *OR* = 0.86 [0.81, 0.91]; EVI_500m_: *OR* = 0.86 [0.81, 0.90]), businessman (NDVI_500m_: *OR* = 0.82 [0.68, 0.99]; EVI_500m_: *OR* = 0.83 [0.69, 0.99]), other laborers (NDVI_500m_: *OR* = 0.77 [0.68, 0.88]; EVI_500m_: *OR* = 0.80 [0.71, 0.91]), and non-smokers (NDVI_500m_: *OR* = 0.77 [0.70, 0.85]; EVI_500m_: *OR* = 0.77 [0.70, 0.85]) showed stronger associations (Table 3).

**Table 3:** Associations between residential greenness exposure and MetS risk stratified by potential modifiers.

**Subgroup**	**NDVI**_**500m**_		**EVI**_**500m**_	
	***OR* (95% *CI*)^a^**	***P*-value for interaction**	***OR* (95% *CI*)^a^**	***P*-value for interaction**
Age, years				
< 60	0.86 (0.81, 0.92)^**^		0.85 (0.79, 0.90)^**^	
≥ 60	1.05 (0.96, 1.15)	< 0.001	1.06 (0.97, 1.16)	< 0.001
Sex				
Female	1.00 (0.94, 1.07)		0.99 (0.92, 1.05)	
Male	0.73 (0.67, 0.80)^**^	< 0.001	0.74 (0.68, 0.81)^**^	< 0.001
Ethnicity				
Han	0.90 (0.85, 0.95)^**^		0.89 (0.85, 0.94)^**^	
Minority	0.90 (0.65, 1.23)	0.50	0.99 (0.74, 1.32)	0.94
Educational level				
Illiteracy	1.01 (0.93, 1.09)		0.98 (0.91, 1.06)	
Primary school or above	0.81 (0.74, 0.89)^**^	< 0.001	0.84 (0.76, 0.92)^**^	< 0.001
Marriage status				
Married/Cohabitation	0.86 (0.81, 0.91) ^**^		0.86 (0.81, 0.90)^**^	
Unmarried	1.25 (0.95, 1.64)	0.11	1.18 (0.90, 1.56)	0.23
Widowed/Divorced/Separated	1.29 (1.11, 1.49)^**^	0.001	1.28 (1.1, 1.48)^**^	0.001
Occupation				
Unemployed	0.94 (0.87, 1.02)		0.92 (0.85, 0.99)	
Famer	1.04 (0.92, 1.16)	0.55	1.04 (0.93, 1.17)	0.46
Businessman	0.82 (0.68, 0.99)^*^	0.04	0.83 (0.69, 0.99)^*^	0.05
Administrative staff	0.79 (0.61, 1.02)	0.07	0.85 (0.66, 1.10)	0.22
Student	1.25 (0.45, 3.47)	0.66	1.27 (0.47, 3.44)	0.63
Other laborers	0.77 (0.68, 0.88)^**^	< 0.001	0.80 (0.71, 0.91)	0.001
Insurance type				
No insurance	0.94 (0.61, 1.45)		0.90 (0.58, 1.39)	
Non-commercial insurance	0.96 (0.62, 1.49)	0.85	0.99 (0.64, 1.54)	0.97
Commercial insurance	0.61 (0.36, 1.02)	0.06	0.61 (0.36, 1.03)	0.07
Drinking status				
Current or former drinkers	0.92 (0.84, 1.01)		0.94 (0.85, 1.03)	
Non-drinkers	0.87 (0.82, 0.92)^*^	0.08	0.84 (0.80, 0.90)	0.20
Smoking status				
Current or former smokers	0.94 (0.89, 1.00)^*^		0.93 (0.88, 0.99)^*^	
Non-smokers	0.77 (0.70, 0.85)^**^	< 0.001	0.77 (0.70, 0.85)^**^	< 0.001
Residential location				
Rural	0.89 (0.84, 0.94)^**^		0.88 (0.83, 0.93)^**^	
Urban	0.88 (0.77, 0.99)^*^	0.04	0.91 (0.80, 1.03)	0.13

CI, confidence interval; EVI, enhanced vegetation index; MetS, metabolic syndrome; NDVI, normalized difference vegetation index; OR, odds ratio.

a Adjusted for age, sex, residential location, ethnicity, educational level, occupation, marriage status, insurance type, diet, smoking status, alcohol drinking status, temperature, and humidity (except for the one used for stratification).

* *P*-value < 0.05;

** *P*-value < 0.01.

### Pathways between greenness exposure and MetS risk

The joint effect of PM_2.5_, PM_10_, NO_2_, SO_2_, PA, and BMI mediated 48.10% and 44.61% of the total effect of NDVI_500m_ and EVI_500m_ on the MetS risk, respectively. Moreover, the independent mediation analysis showed that PM_10_ (accounting for 22.04% of the total effect), NO_2_ (24.12%), and BMI (24.97%) significantly mediated the association between NDVI_500m_ and MetS risk. The most substantial mediation effect was observed in BMI (24.97%). A similar mediation effect was observed for PM_10_, NO_2_, and BMI on the association of EVI_500m_ with MetS risk (PM_10_, NO_2_, and BMI accounting for 20.90%, 21.89%, and 25.60% of the total effect, respectively) (Table 4).

**Table 4:** Mediation of associations between residential NDVI_500m_ and EVI_500m_ and MetS risk^a^.

	**NDVI**_**500m**_		**EVI**_**500m**_	
**Mediator**	**Indirect effects,** ** β % (95% *CI*)^a^**	**Proportion mediated %(95% *CI*)^a^**	**Indirect effects** ** β % (95% *CI*)^a^**	**Proportion mediated %(95% *CI*)^a^**
PM_2.5_	−0.02 (−0.03, 0.02)	4.09 (−3.11, 11.44)	−0.02 (−0.05, 0.01)	3.84 (−0.39, 14.34)
PM_10_	−0.08 (−0.13, −0.03)^*^	22.04 (10.91, 68.22)^*^	−0.10 (−0.18, −0.05)^*^	20.90 (8.70, 34.40)^*^
NO_2_	−0.09 (−0.12, −0.07)^*^	24.12 (14.90, 39.20)^*^	−0.11 (−0.14, −0.09)^*^	21.89 (16.97, 35.44)^*^
SO_2_	0.00 (0.00, 0.01)	0.10 (−1.80, 1.35)	0.00 (0.00, 0.00)	0.10 (−0.71, 0.83)
PA	0.00 (0.00, 0.01)	−1.04 (−2.49, −0.02)	0.01 (0.00, 0.01)	−1.06 (−1.85, 0.23)
BMI	−0.09 (−0.11, −0.07)^*^	24.97 (18.62, 39.86)^*^	−0.12 (−0.15, −0.10)^*^	25.60 (17.82, 58.53)^*^
**Joint**	−0.17 (−0.22, −0.11)^*^	48.10 (32.41, 92.08)^*^	−0.21 (−0.30, −0.13)^*^	44.61 (24.49, 95.49)^*^

BMI, body mass index; EVI, enhanced vegetation index; MetS, metabolic syndrome; NDVI, normalized difference vegetation index; NO_2_, nitrogen dioxide; PM_2.5_, particles with aerodynamic diameter ≤ 2.5 μm; PM_10_, particulate matter with an aerodynamic diameter ≤ 10 μm; PA, Physical activity; SO_2_, sulfur dioxide.

a Adjusted for age, sex, ethnicity, educational level, occupation, marriage status, residential location, diet, smoking status, alcohol drinking status, temperature, and humidity.

* *P*-value < 0.01.

## Discussion

Our study found that long-term exposure to residential greenness was associated with the risk for MetS, and the associations were independently mediated by PM_10_, NO_2_, and BMI. The joint mediation effect of PM_2.5_, PM_10_, NO_2_, SO_2_, PA, and BMI was as high as 48.10% and 44.61% of the total effect of NDVI_500m_ and EVI_500m_ on the MetS risk, respectively. The findings provided substantial evidence on the effects of residential greenness and its medicated mechanism on the MetS risk. This evidence would raise attention to neighborhood greenness for reducing the chronic disease burden in China.

We observed the negative association between residential greenness exposure and the MetS risk in this study. To the best of our knowledge, two previous studies explored the relationship between long-term exposure to greenness and the incidence or prevalence of MetS, but their results were inconsistent (15, 39). For example, a cross-sectional study indicated a beneficial association between residential greenness and MetS in the Chinese urban population (15). However, another study using multi-exposure models showed that greenness exposure was inverse but non-significant associated with prevalent and incident MetS (39). We observed an inverse association between greenness exposure and risk of MetS with adjustment for all covariates, including demographic characteristics, lifestyle, and environmental factors. This relationship showed that the lack of adjustments for these covariates may underestimate the association. Therefore, applying our findings to other study areas or populations would solely depend on the correlation between residential greenness, demographic characteristics, and lifestyle factors. However, our findings suggested a positive association of EVI_500m_ with reduced HDL-cholesterol, which was inconsistent with a previous study that greenness had a negative association with reduced HDL-cholesterol (39, 40). The possible reason may be explained by the cross-sectional study design, and high disparities across the different populations. Further prospective studies are needed to explore the association between greenness exposure and blood lipid levels.

Air pollutants and BMI significantly mediated the association between residential greenness exposure and MetS risk. There are several proposed hypotheses for the beneficial effects of greenness exposure. First, exposure to higher concentrations of air pollutants, such as PM_2.5_, PM_10_, NO_2_, and SO_2_, was associated with an elevated risk of MetS. Greenness exposure could attenuate the adverse effect of air pollution exposure by removing particulate matter and carbon monoxide (41, 42). Our study found that the PM_10_ and NO_2_ could mediate about 20.90%–24.12% of the association between NDVI_500m_ / EVI_500m_ and MetS risk. In contrast, we did not find a significant mediation effect between PM_2.5_ and SO_2_. A possible explanation is that PM_10_ and NO_2_ in our study site were more serious, while the concentration of PM_2.5_ and SO_2_ may be relatively low (43). Besides, PM_10_ has a larger particle size and concentration than PM_2.5_, PM_10_ is more likely to be dry deposited on vegetation surfaces through gravity sedimentation. Once particles are impacted onto the vegetation surface, some particulates could be washed away by rain and transferred to the soil, re-suspended in the atmosphere by wind, or fall to the ground with leaves and branches (44, 45). Thus, plants may absorb more PM_10_ than PM_2.5_. One previous study also reported similarities, indicating that only PM_10_, NO_2_, and O_3_ had mediating effects (15). Second, BMI is a leading risk factor for MetS (31), and high community greenness exposure is reported to be associated with reduced obesity risk (46). People living with high greenness exposure are more likely to participate in regular physical activity and be associated with lower BMI (47). A previous study in China showed that physical activity could mediate about 50% of the association between NDVI and hypertension (48). In hindsight, our study found that BMI mediated the association but not PA. Therefore, participants with a history of MetS-related components would have a modified physical activity pattern as a non-pharmacological intervention; and their BMI would change slower. Another possible explanation is that BMI was calculated by objective measurements, whereas PA was self-reported by participants, which may induce recall bias. Besides, these mediators may interact with each other and have a role in human health. Our results by multiple mediation analysis indicated that the joint mediation effect of PM_2.5_, PM_10_, NO_2_, SO_2_, PA, and BMI could explain almost half of the association. The sensitivity analysis demonstrated that these associations were stable.

In subgroup analysis, we observed that the association between greenness exposure and MetS risk was more substantial in males, participants aged < 60 years, those without smoking habits, an educational level of primary school or above, and being married/cohabitation, other laborers, and businessman. A previous study has also explored the age-specific effects of greenness on MetS, indicating that participants aged < 65 years had a more substantial effect estimate (46). Perhaps, younger adults are more likely to use the outdoor green space than older people and can benefit more from greenness exposure. The sex-related biological (e.g., sex hormones and immune and inflammation responses), occupational (e.g., outdoor physical activity), and lifestyle factors (e.g., smoking and drinking habits) may result in sex disparities in the greenness exposure effect (49). Married, better-educated people and businessmen usually have more social support and social resources, and are able to live in communities with better environments and thus benefit more from greenness exposure (44). In addition, the association of non-smoking habits with more healthy lifestyle habits, including greenness in residential environments, can be said to link and influence one another.

To our knowledge, this study has at least two major strengths. First, the stratified cluster random sampling method adopted in this study better represents the heterogeneity of population and environmental exposure. It would also increase the generalizability of our findings. Second, both single and multiple mediation analyses were used to estimate the indirect effects of the selected mediators. This combination of mediation analyses led to the sufficient statistical power to detect modest effects and help deeply understand the potential mechanisms underlying the association between residential greenness and the MetS risk.

There are several avenues for potential improvement or further exploration of this study. First, as the MetS were only measured once, our research design could not fully reveal a causal relationship between long-term residential greenness exposure and MetS risk. However, the estimation of greenness exposure was the average exposure level for the past three years, which reduced the chance of reverse causality. Second, although we employed EVI and NDVI as proxies of the overall level of residential greenness, there are limitations in information quality and accuracy given the nature of the satellite imagery data. Moreover, the satellite-derived information of urban greenness might not fully capture all pathways through which humans experience nature. More could be done in the future when other micro-level environmental auditing approaches became available, and a follow-up analysis of the eye-level based visibility of greenness could be done to enhance and validate the current work. Lastly, the health effect of residential greenness exposure may vary depending on daily time-activity patterns. However, these unmeasured activity patterns may affect the effect estimates. Hence, we suggest that wearable devices, such as a smartwatch, be used in a nested small sample of our study. This move would precisely measure daily time-activity patterns and, thus, help further calibrate individual exposures at the population level.

## Conclusions

This large-scale epidemiological study provides a better understanding of the impact of residential greenness exposure on MetS risk among adults in southeastern China. Such impact was found to be especially apparent in young adults, males, and those without smoking habits. PM_10_, NO_2_, and BMI mediated the association between residential greenness exposure and MetS, and the joint mediation effect of PM_2.5_, PM_10_, NO_2_, SO_2_, PA, and BMI may explain almost half of the association.

This study has highlighted the importance of residential greenness exposure to reduce the MetS risk and thereby the potential for use as a framework for improving public health in urban areas. Multiple stakeholders, such as policymakers, researchers, and urban planners, can benefit from the findings of this study. The evidence would serve for the optimization of environmental protection policies and the development of healthier cities.

We suggest that human health outcomes should be incorporated into the goals of green space related urban design, planning, and policy-making. Our study makes the connection between urban greenness and health outcomes more visible, which will facilitate and encourage collaborations across different sectors, including epidemiology, public health, urban planning, and municipal and civil engineering. The results of the current study will further safeguard green spaces in the city as an important asset for supporting health and wellbeing. It will also facilitate healthier urban design and development in the future and thereby getting a true long-term legacy.

## Data availability statement

The original contributions presented in the study are included in the article/Supplementary material, further inquiries can be directed to the corresponding authors.

## Ethics statement

The Ethical Review Board of the Fujian Provincial Center for Disease Control and Prevention reviewed and approved the study protocol (2018001). The participants provided their written informed consent to participate in this study.

## Author contributions

XiaL, QW, and SY was responsible for data cleaning. WZ and XiaL was responsible for obtaining funding. SY, WZ, PJ, and XiaL were responsible for study design. SY contributed in revision. PJ, XiaL, QW, and SY were responsible for manuscript preparation. QW, SY, WZ, PJ, and XiaL were responsible for methodology. PX, CF, BY, YF, SD, GQ, DJ, XiaL, QW, SY, and NL were responsible for data cleaning. XiL, YY, SH, XiuL, YZ, WZ, XiaL, and XF were responsible for data collection. All authors contributed to the article and approved the submitted version.
