# Supplementary material for: Associations and pathways between residential greenness and metabolic syndromes in Fujian Province
Source: Front Public Health. 2022 Dec 22;10:1014380. doi: 10.3389/fpubh.2022.1014380 (PMC9815145; doi:10.3389/fpubh.2022.1014380)
Supplement: Supplementary file 1 [file Data_Sheet_1.docx]

**Supplemental material**

**Figure S1.** A flowchart of sampling process of the participants

**Figure S2.** A flowchart of participant inclusion and exclusion

**Figure S3.** A directed acyclic graph for the association between greenness and metabolic syndrome

**Figure S4.** Correlations between different greenness indexes

**Table S1.** Sensitivity analysis for the association of each interquartile range (IQR) increase in NDVI_250m_ and EVI_250m_ with MetS

**Table S2.** Sensitivity analysis for the association of each interquartile range (IQR) increase in NDVI_1000m_ and EVI_1000m_ with MetS

**Table S3.** Sensitivity analysis for the association of each interquartile range (IQR) increase in NDVI and EVI over different exposure times with MetS


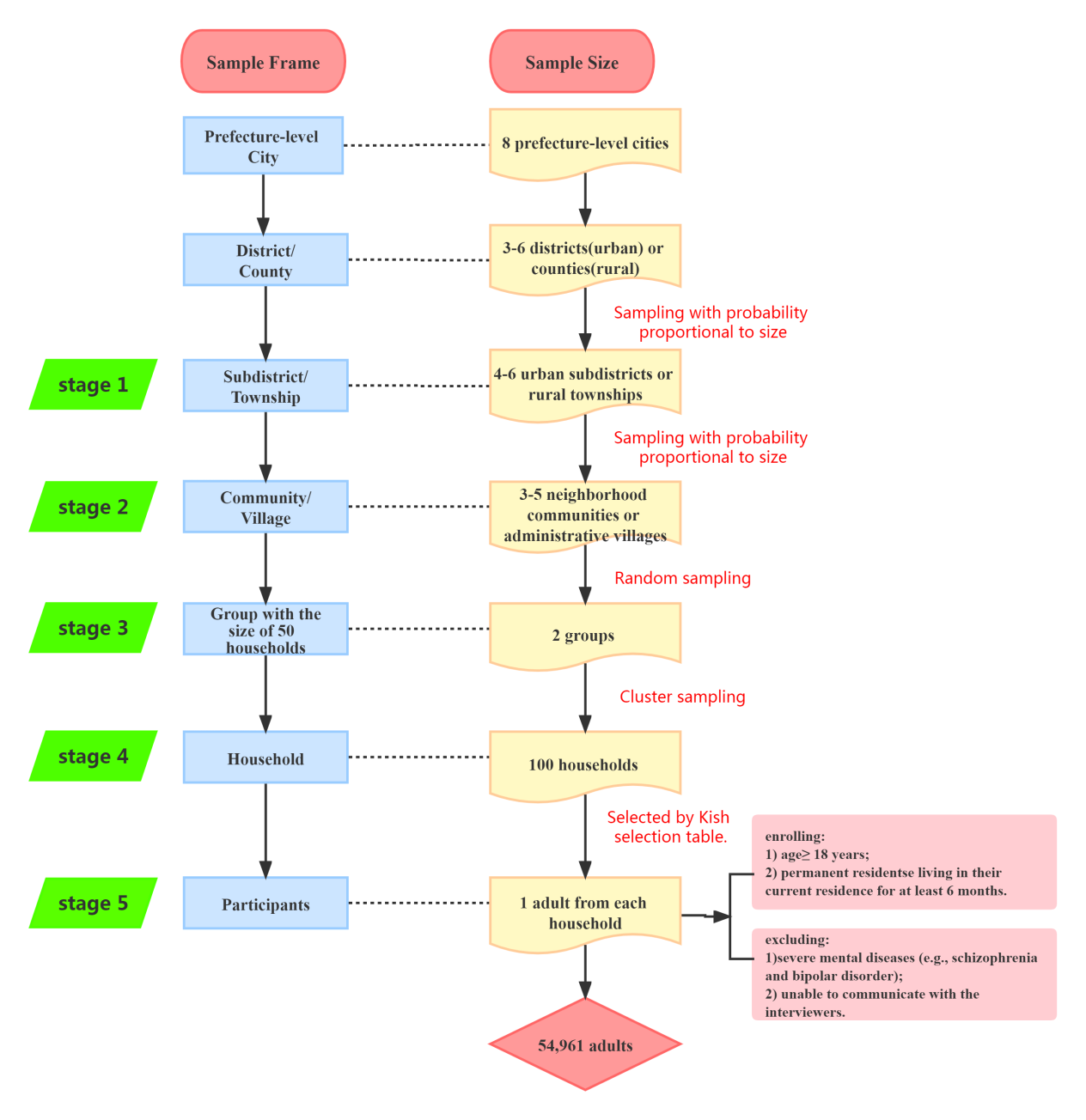


**Figure S1.** A flowchart of participants sampled process


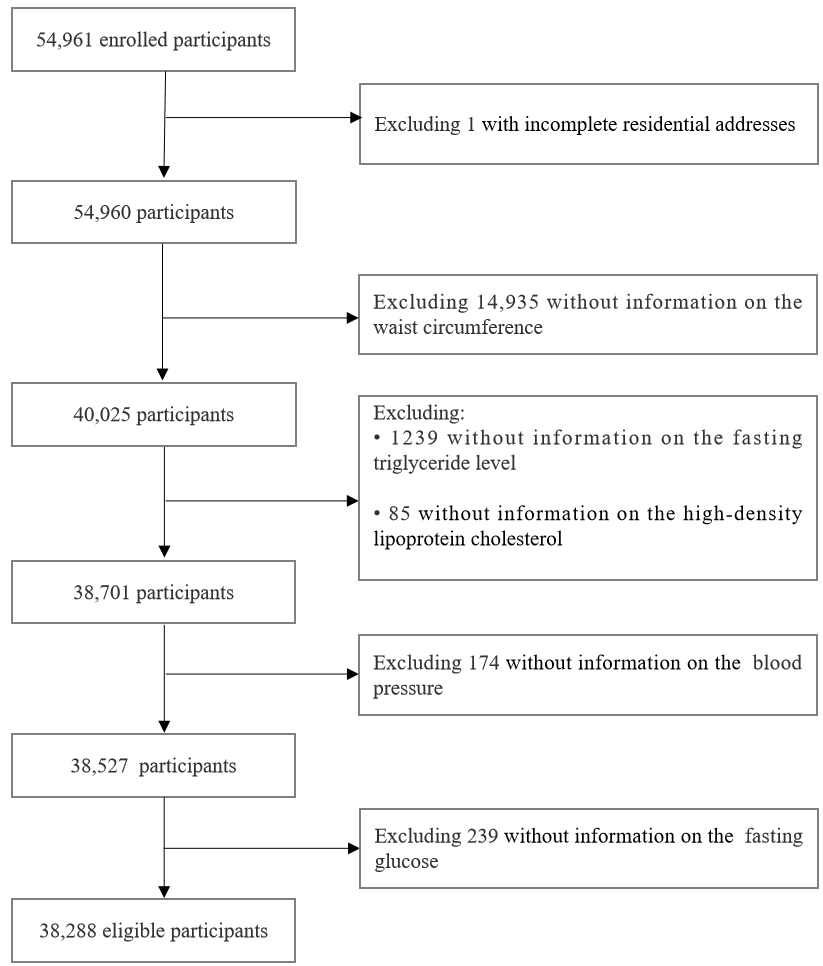


**Figure S2.** A flowchart of participant enrolment


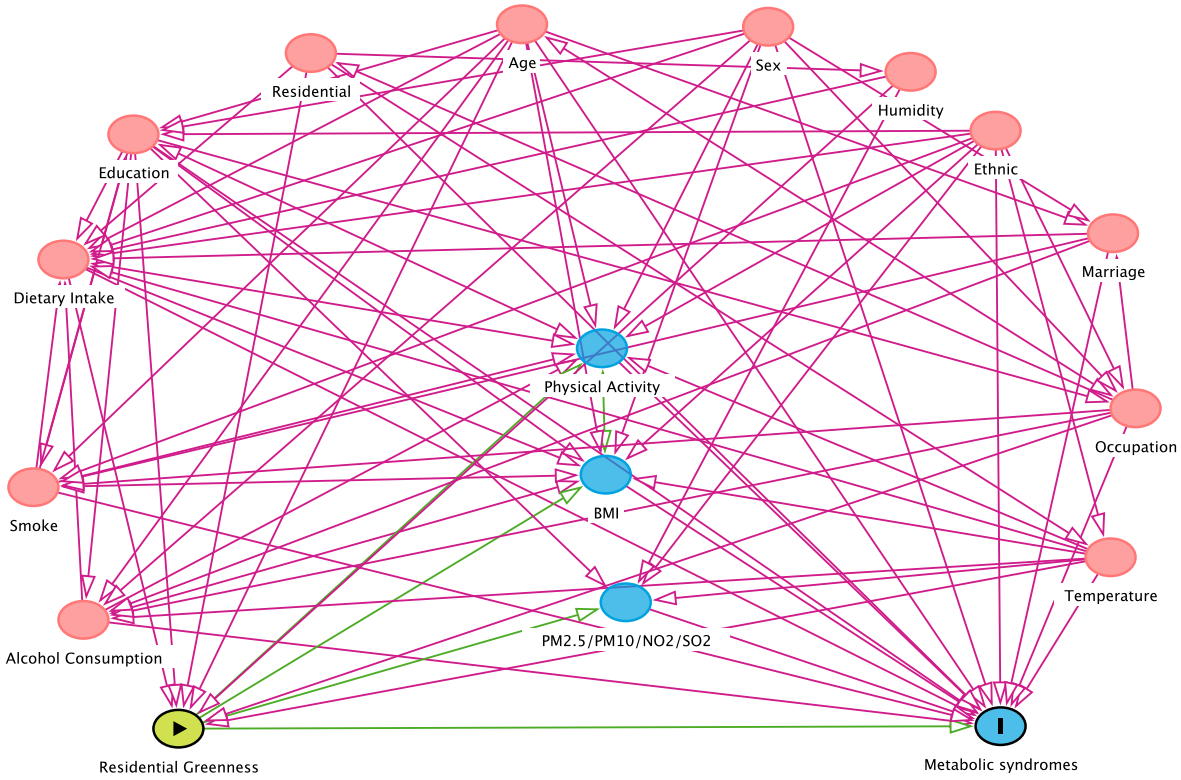


**Figure S3. Directed acyclic graph for the associations between greenness and metabolic syndromes**, showing potential confounders and mediators. Pink lines indicate potential confounders, and green lines indicate potential mediators.

NO_2_, nitrogen dioxide; PM_2_._5_, particle with aerodynamic diameter ≤2.5 µm; PM_10_, particle with aerodynamic diameter ≤10 µm; SO_2_, sulfur dioxide.


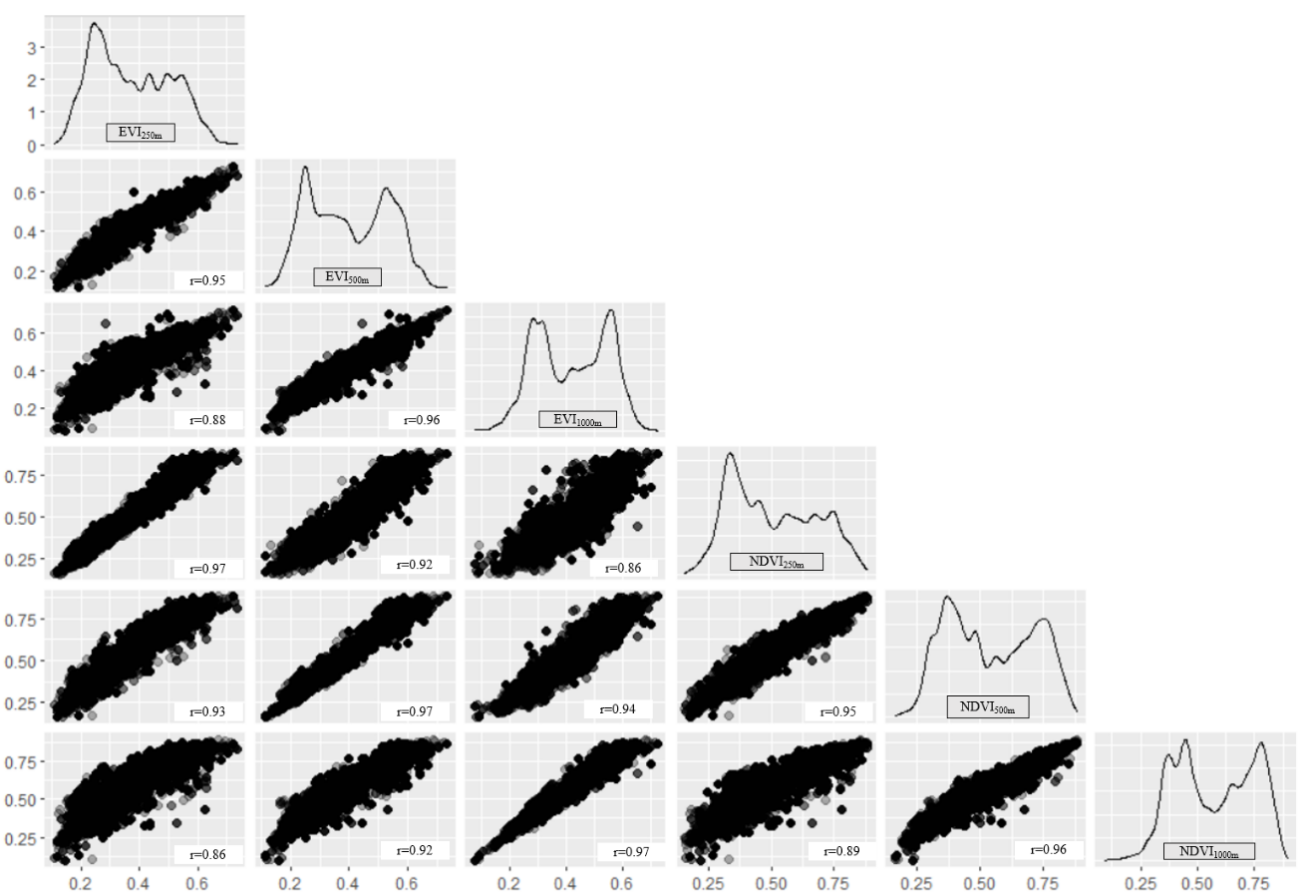


**Figure S4. Correlations between the different greenness**

**Table S1. Sensitivity analysis for the associations of each IQR increase in NDVI_250m_ and EVI_250m_ with MetS risk**

|  | *OR* (95%*CI*) for **NDVI_250m_** | | *OR* (95%*CI*) for **EVI_250m_** | |
| --- | --- | --- | --- | --- |
| MetS related indexes | Crude model | Adjusted model ^a^ | Crude model | Adjusted model ^a^ |
| **MetS** | 1.05 (1.01, 1.10)^*^ | 0.89 (0.84, 0.93)^**^ | 1.01 (0.97, 1.05)^**^ | 0.88 (0.84, 0.92)^***^ |
| Central obesity | 0.59 (0.45, 0.78)^**^ | 0.70 (0.52, 0.96)^*^ | 0.65 (0.50, 0.85)^*^ | 0.76 (0.57, 1.02) |
| Elevated triacylglyceride | 1.05 (1.01, 1.09)^*^ | 0.93 (0.90, 0.97)^**^ | 1.01 (0.97, 1.04) | 0.92 (0.89, 0.96)^***^ |
| Reduced HDL-cholesterol | 1.07 (1.03, 1.11) | 1.05 (1.00, 1.10) | 1.05 (1.01, 1.10)^*^ | 1.05 (1.01, 1.10)^*^ |
| High blood pressure | 1.20 (1.16, 1.24)^**^ | 0.97 (0.93, 1.01) | 1.17 (1.13, 1.21)^***^ | 0.96 (0.93, 1.00) |
| Raised fasting glucose | 0.83 (0.80, 0.86)^**^ | 0.69 (0.66, 0.72)^**^ | 0.80 (0.78, 0.84)^***^ | 0.69 (0.66, 0.72)^***^ |

EVI: enhanced vegetation index; IQR: interquartile range; MetS: metabolic syndrome; NDVI: normalized difference vegetation index.

^a^Adjusted for age, sex, ethnicity, educational level, occupation, marriage status, residential location, diet, smoking status, alcohol drinking status, temperature, and humidity.

^*^*P*<0.05; ^**^*P*<0.01; ^***^*P*<0.001

**Table S2. Sensitivity analysis for the associations of each IQR increase in NDVI_1000m_ and EVI_1000m_ with MetS**

|  | *OR* (95%*CI*) for **NDVI_1000m_** | | *OR* (95%*CI*) for **EVI_1000m_** | |
| --- | --- | --- | --- | --- |
| MetS related indexes | Crude model | Adjusted model ^a^ | Crude model | Adjusted model ^a^ |
| **MetS** | 1.11 (1.07, 1.16)^**^ | 0.89 (0.84, 0.94)^**^ | 1.03 (0.99, 1.06) | 0.89 (0.85, 0.94)^**^ |
| Central obesity | 0.51 (0.39, 0.67)^**^ | 0.59 (0.43, 0.82)^**^ | 0.57 (0.43, 0.75)^**^ | 0.68 (0.50, 0.92)^*^ |
| Elevated triacylglyceride | 1.13 (1.08, 1.17)^**^ | 0.98 (0.93, 1.02) | 1.05 (1.01, 1.08)^*^ | 0.98 (0.94, 1.01) |
| Reduced HDL-cholesterol | 1.06 (1.02, 1.11)^*^ | 1.00 (0.95, 1.05) | 1.05 (1.01, 1.08)^*^ | 1.03 (0.99, 1.07) |
| High blood pressure | 1.27 (1.23, 1.32)^**^ | 1.00 (0.95, 1.04) | 1.17 (1.13, 1.21)^**^ | 0.99 (0.95, 1.02) |
| Raised fasting glucose | 0.89 (0.86, 0.93)^**^ | 0.70 (0.67, 0.74)^**^ | 0.87 (0.84, 0.90)^**^ | 0.72 (0.69, 0.76)^**^ |

EVI: enhanced vegetation index; IQR: interquartile range; MetS: metabolic syndrome; NDVI: normalized difference vegetation index.

^a^Adjusted for age, sex, ethnictiy, educational level, occupation, marriage status, residential location, diet, smoking status, alcohol drinking status, temperature, and humidity.

^*^*P*<0.01; ^**^*P*<0.001

**Table S3.** Sensitivity analysis for the associations of each IQR increase in NDVI and EVI in different exposure times with MetS

|  | ***OR* (95%*CI*)** ^a^ | | | | | |
| --- | --- | --- | --- | --- | --- | --- |
| Greenness  exposure times | **MetS** | Central obesity | Elevated triacylglyceride | Reduced HDL cholesterol | High blood  pressure | Raised fasting  glucose |
| **NDVI_250m_** |  |  |  |  |  |  |
| One year | 0.88 (0.84, 0.92)^***^ | 0.71 (0.52, 0.96)^*^ | 0.96 (0.93, 1.00) | 1.00 (0.96, 1.05) | 0.97 (0.93, 1.01) | 0.69 (0.67, 0.72)^***^ |
| Two years | 0.88 (0.84, 0.92)^***^ | 0.71 (0.52, 0.97)^*^ | 0.97 (0.93, 1.01) | 1.01 (0.97, 1.05) | 0.97 (0.94, 1.01) | 0.69 (0.66, 0.72)^***^ |
| Three years | 0.89 (0.84, 0.93)** | 0.70 (0.52, 0.96)* | 0.93 (0.90, 0.97)** | 1.05 (1.00, 1.10) | 0.97 (0.93, 1.01) | 0.69 (0.66, 0.72)** |
| **NDVI_500m_** |  |  |  |  |  |  |
| One year | 0.86 (0.82, 0.91)^***^ | 0.62 (0.44, 0.87)^**^ | 0.96 (0.92, 1.00) | 1.01 (0.96, 1.06) | 0.99 (0.94, 1.03) | 0.66 (0.63, 0.69)^***^ |
| Two years | 0.87 (0.82, 0.91)^***^ | 0.63 (0.45, 0.88)^**^ | 0.97 (0.93, 1.01) | 1.01 (0.97, 1.06) | 0.99 (0.95, 1.04) | 0.66 (0.63, 0.69)^***^ |
| Three years | 0.87 (0.83, 0.92)*** | 0.66 (0.48, 0.91)* | 0.93 (0.89, 0.98)* | 1.04 (0.99, 1.09) | 0.97 (0.93, 1.02) | 0.68 (0.65, 0.71)*** |
| **NDVI_1000m_** |  |  |  |  |  |  |
| One year | 0.88 (0.83, 0.93)^***^ | 0.58 (0.41, 0.82)^**^ | 1.00 (0.95, 1.04) | 0.98 (0.93, 1.03) | 1.01 (0.97, 1.06) | 0.66 (0.63, 0.70)^***^ |
| Two years | 0.88 (0.83, 0.93)^***^ | 0.56 (0.40, 0.80)^**^ | 1.00 (0.96, 1.05) | 0.98 (0.93, 1.03) | 1.02 (0.97, 1.07) | 0.67 (0.63, 0.70)^***^ |
| Three years | 0.89 (0.84, 0.94)*** | 0.59 (0.43, 0.82)*** | 0.98 (0.93, 1.02) | 1.00 (0.95, 1.05) | 1.00 (0.95, 1.04) | 0.70 (0.67, 0.74)*** |
| **EVI_250m_** |  |  |  |  |  |  |
| One year | 0.88 (0.84, 0.92)^***^ | 0.75 (0.56, 1.01) | 0.94 (0.90, 0.97)^***^ | 1.04 (0.99, 1.08) | 0.97 (0.94, 1.02) | 0.70 (0.68, 0.73)^***^ |
| Two years | 0.89 (0.85, 0.93)^***^ | 0.74 (0.55, 1.01) | 0.94 (0.90, 0.98)^*^ | 1.05 (1.00, 1.09) | 0.98 (0.94, 1.02) | 0.70 (0.67, 0.73)^***^ |
| Three years | 0.88 (0.84, 0.92)*** | 0.76 (0.57, 1.02) | 0.92 (0.89, 0.96)*** | 1.05 (1.01, 1.10)* | 0.96 (0.93, 1.00) | 0.69 (0.66, 0.72)*** |
| **EVI_500m_** |  |  |  |  |  |  |
| One year | 0.87 (0.83, 0.91)^***^ | 0.71 (0.52, 0.97)^*^ | 0.94 (0.90, 0.98)^*^ | 1.04 (0.99, 1.09) | 0.97 (0.93, 1.02) | 0.68 (0.65, 0.71)^***^ |
| Two years | 0.88 (0.83, 0.92)^***^ | 0.68 (0.49, 0.95)^*^ | 0.94 (0.90, 0.98)^*^ | 1.04 (0.99, 1.09) | 0.99 (0.94, 1.03) | 0.68 (0.65, 0.71)^***^ |
| Three years | 0.87 (0.82, 0.91)*** | 0.76 (0.56, 1.04) | 0.91 (0.87, 0.95)*** | 1.06 (1.01, 1.11)* | 0.96 (0.92, 1.00) | 0.69 (0.66, 0.72)*** |
| **EVI_1000m_** |  |  |  |  |  |  |
| One year | 0.88 (0.84, 0.93)^***^ | 0.61 (0.44, 0.84)^**^ | 0.99 (0.95, 1.03) | 1.01 (0.96, 1.06) | 1.00 (0.95, 1.04) | 0.68 (0.65, 0.72)^***^ |
| Two years | 0.89 (0.85, 0.94)^***^ | 0.59 (0.42, 0.83)^**^ | 0.98 (0.94, 1.03) | 1.00 (0.95, 1.06) | 1.01 (0.97, 1.06) | 0.70 (0.67, 0.73)^***^ |
| Three years | 0.89 (0.85, 0.94)*** | 0.68 (0.50, 0.92)** | 0.98 (0.94, 1.01) | 1.03 (0.99, 1.07) | 0.99 (0.95, 1.02) | 0.72 (0.69, 0.76)*** |

EVI: enhanced vegetation index; IQR: interquartile range; MetS: metabolic syndrome; NDVI: normalized difference vegetation index.

^a^Adjusted for age, sex, ethnicity, educational level, occupation, marriage status, residential location, diet, smoking status, alcohol drinking status, temperature ,and humidity.

^*^*P*<0.05; ^**^*P*<0.01; ^***^*P*<0.001
